# Biopolymer‐based Carriers for DNA Vaccine Design

**DOI:** 10.1002/anie.202010282

**Published:** 2021-01-07

**Authors:** Christoph O. Franck, Luise Fanslau, Andrea Bistrovic Popov, Puneet Tyagi, Ljiljana Fruk

**Affiliations:** ^1^ Department of Chemical Engineering and Biotechnology University of Cambridge Phillipa Fawcett Drive Cambridge CB3 0AS UK; ^2^ Dosage Form Design and Development BioPharmaceuticals Development R&D Astra Zeneca Gaithersburg MD 20878 USA

**Keywords:** biopolymers, DNA vaccines, gene delivery, immunotherapy, nanostructure

## Abstract

Over the last 30 years, genetically engineered DNA has been tested as novel vaccination strategy against various diseases, including human immunodeficiency virus (HIV), hepatitis B, several parasites, and cancers. However, the clinical breakthrough of the technique is confined by the low transfection efficacy and immunogenicity of the employed vaccines. Therefore, carrier materials were designed to prevent the rapid degradation and systemic clearance of DNA in the body. In this context, biopolymers are a particularly promising DNA vaccine carrier platform due to their beneficial biochemical and physical characteristics, including biocompatibility, stability, and low toxicity. This article reviews the applications, fabrication, and modification of biopolymers as carrier medium for genetic vaccines.

## Graphical abstract

Recently, the first genetic vaccine has been approved to tackle the COVID‐19 pandemic. Despite their favourable properties, low transfection efficacies prevent many nucleic acid vaccines from being approved for human use. This Review explores strategies that use biopolymeric delivery systems to improve the efficacy of DNA immunisation.

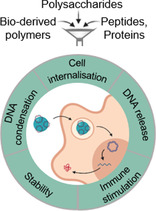

## 1 DNA vaccines

The development of vaccines has been one of the most significant advances of modern medicine and has led to improved public health and life expectancy. It has been reported that two to three million lives per year are saved worldwide due to vaccination.^[1]^ The process of immunisation was first observed in China and the Middle East in the 12^th^ century. By using the skin or pustule liquid of patients with smallpox, disease resistance in another patient was achieved.[^2^, ^3^] However, the first vaccine was developed in 1798 by Edward Jenner who reported that injecting humans with cowpox led to the protection against smallpox.^[4]^ Conventional vaccines are predominantly composed of inactivated (killed) pathogens or pathogen subunits, for example toxins, polysaccharides or proteins, and live‐attenuated (weakened) viruses.^[5]^ Antigens from these pathogens are recognised by the immune system as being foreign, which results in the induction of an immune response, the production of antibodies, and the establishment of immunological memory. Successful traditional vaccines have been developed against numerous bacterial and viral pathogens and have been most effective for disease control.^[6]^

**Figure 1:**
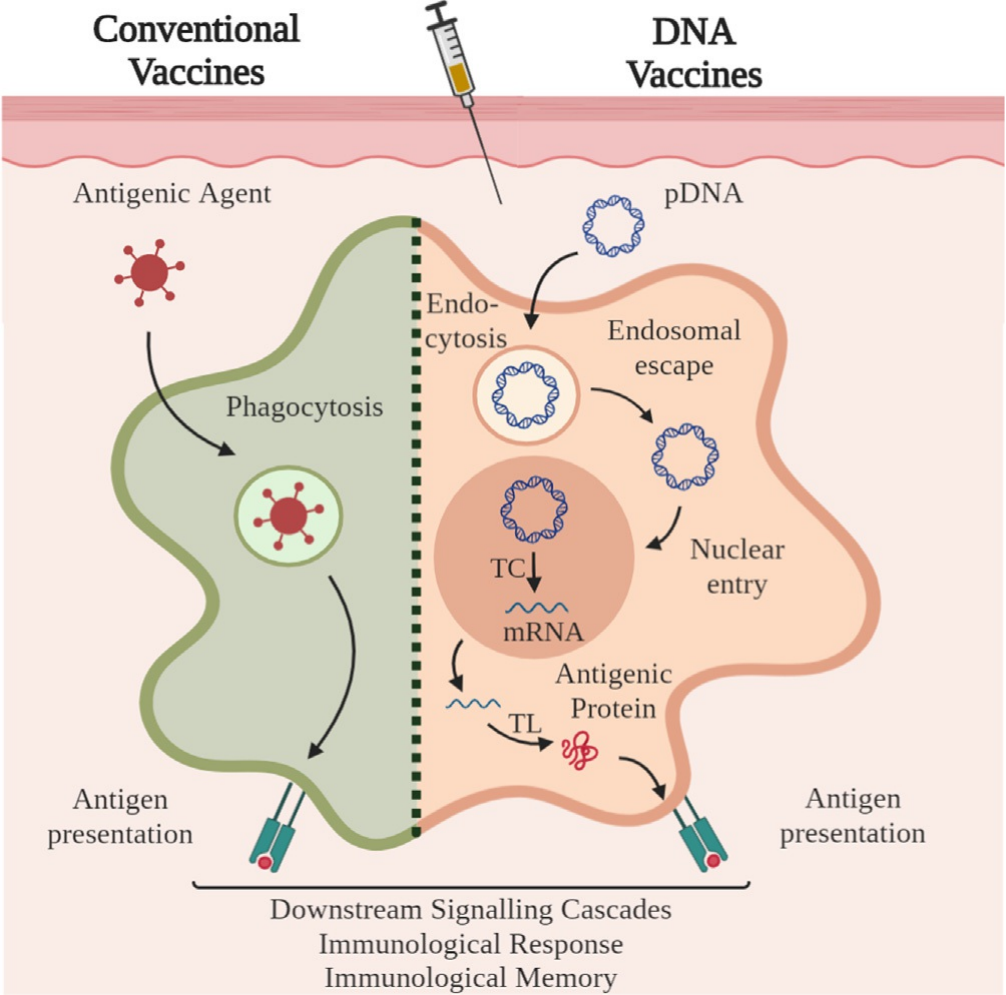
Difference between conventional and DNA vaccines. Conventional vaccines (left): After processing of the antigenic agent (e.g. inactivated pathogens, pathogen subunits, live‐attenuated pathogens), the antigen is presented to the immune system by antigen‐presenting cells (APCs). DNA vaccines (on the right): The administered plasmid DNA (pDNA) is endocytosed, released in the cytosol and taken up by the nucleus, followed by transcription and translation into an antigenic protein. This agent is presented to other cells of the immune system. For both pathways, the antigen presentation results in downstream signalling cascades evoking an immunological response and leading to the establishment of an immunological memory.

Unlike conventional vaccines, DNA vaccines are bacterial plasmids designed to carry a specific encoding gene, which is responsible for expression of the desired antigen in the host and leads to the induction of an immune response.[^7^, ^8^] Instead of using proteins from pathogens to stimulate the immune system, DNA vaccines deliver an instruction for the protein to be produced in the body (Figure 1). Major benefits of DNA vaccines, compared to conventional vaccines, are high specificity and the possibility of introducing additional sequences to the plasmid, for example, adjuvants, which can potentiate the immunostimulatory effect of the expressed antigen.^[9]^ In addition, the expressed immunising antigen is exposed to the same species‐specific post‐translational modifications (e.g. phosphorylation or glycosylation) as the natural viral infection. There is no need for live vectors or complex biochemical production strategies. The interest in DNA vaccines was further boosted by promising animal studies, and generally, cheaper production as well as facile transport and storage.

So far, DNA vaccines have been approved for veterinary use against West Nile Virus in horses,^[10]^ canine melanoma,^[11]^ infectious haematopoietic necrosis in farm‐raised Atlantic salmon,^[12]^ and as gene therapy for growth hormone‐releasing hormone in pigs.^[13]^ The first human trial of DNA‐based vaccines was conducted in 1998 and targeted human immunodeficiency virus type 1 (HIV).^[14]^ Currently, there are many ongoing human clinical trials that target various types of cancer, autoimmune diseases, and infectious diseases, such as human papilloma virus (HPV), HIV, hepatitis B and coronavirus (Table 1).[^5^, ^15^]

**Table 1:** Active clinical trials using DNA‐based vaccines. Data collected from www.clinicaltrials.gov on 14 July 2020.

Therapy focus	Treatment	Disease	Clinical trial phase	NCT number (for identification)
Viral infection vaccines	INO‐4800	Coronavirus	I	NCT04336410
			
	GX‐19—plasmid S‐protein antigen	Coronavirus	I/II	NCT04445389
			
	AG0301	Coronavirus	I/II	NCT04463472
			
	Gn and Gc Hantaan virus plasmid	Hantaan	I	NCT02776761
			
	Interleukin‐12 (IL‐12) DNA	HIV	I/II	NCT03606213
			
	VGX‐3100	HPV	II	NCT03603808
				
Cancer vaccines	pTVG‐HP vaccine + Pembrolizumab	Prostate and metastatic cancer	II	NCT04090528
			
	pTVG‐HP vaccine + nivolumab	Prostate cancer	II	NCT03600350
			
	Neoantigen DNA + nivolumab/pilimumab	Metastatic Prostate Cancer	I	NCT03532217
			
	Neoantigen DNA + durvalumab	Breast cancer	I	NCT03199040
			
	GNOS‐PV02+IL12 + Pembrolizumab	Hepatocellular carcinoma	I/II	NCT04251117
			
	VEGFR‐2 expressing pDNA + Avelumab	Recurrent Glioblastoma	I/II	NCT03750071
			
	Mammagloblin‐A	Breast cancer	I	NCT02204098
			
	pNGVL4a‐Sig/E7(detox)/HSP70 DNA vaccine	Cervical intraepithelial neoplasia	I	NCT00788164
			
	IFx‐Hu2.0 plasmid encoding *S. pyogenes* bacterial antigen Emm55	Cutaneous Melanoma	I	NCT03655756
			
	MEDI0457 + Durvalumab	Oropharynx Cancer	II	NCT04001413
			
	CD105/Yb‐1/SOX2/CDH3/MDM2‐polyepitope pDNA Vaccine	Breast cancer	I	NCT02157051
				
Cancer + viral infection vaccines	HPV E6/E7 DNA vaccine GX‐188E + Pembrolizumab	Cervical cancer, HPV	I/II	NCT03444376
			
	IL‐12/HPV DNA plasmid + Durvalumab	Metastatic melanoma, HPV	II	NCT03439085
			
	pNGVL4a‐Sig/E7(detox)/HSP70 DNA vaccine + Imiquimod	Cervical cancer, HPV	I	NCT00788164

Results of these first clinical trials reported DNA vaccines to be safe and well tolerated, but showed low immunogenicity, which was attributed to insufficient protein expression levels.[^5^, ^11^] This low efficacy can be overcome by optimisation of the plasmid‐encoded antigen to increase antigen expression per cell or by increasing the transfection rate through polymer and lipid formulations, as well as enhancement of the immune response by addition of molecular adjuvants.^[9]^

Notably, mRNA vaccines have recently emerged as another nucleic acid vaccination strategy, as reviewed by Pardi et al.^[16]^ and Maruggi et al.^[17]^ The field of mRNA vaccines gained significant traction and public media attention when BioNTech/Pfizer and Moderna presented the results of Phase 3 clinical trials for their respective mRNA‐based COVID‐19 vaccine candidates in late 2020. On December 2, 2020, the Medicines & Healthcare Products Regulatory Agency (MHRA) granted the worldwide first temporary authorisation for the BioNTech/Pfizer vaccine in the UK, marking an important milestone in the fight against the global coronavirus pandemic and thus making it the first genetic vaccine approved for human use. Both BioNTech/Pfizer and Moderna employ mRNA that encodes for different subunits of the S protein of SARS‐CoV‐2. Major limitations of the technology, e.g. low delivery efficacy and stability concerns in vivo, were overcome using sophisticated nanotechnology. The mRNA is encapsulated in lipid nanoparticle formulations to protect it against rapid nuclease degradation in vivo and deliver it efficiently to the cytoplasm.^[18]^ In contrast to DNA vaccines, mRNA vaccines do not require entry to the nucleus and efficient transcription, thereby excluding the risk of insertional mutagenesis. However, DNA vaccines are known to be generally more stable than mRNA.

Herein, we will give an overview of biopolymer‐based formulations with emphasis on nanostructured biopolymers as carriers for DNA vaccines and introduce modifications that have been shown to enhance vaccine efficiency. Due to the comparable mechanism of action of DNA vaccines and mRNA vaccines, the design considerations presented in this Review may also be useful for mRNA vaccine delivery systems.

### 1.1 DNA Vaccines: Mode of Action

The administration of DNA vaccines leads to the transfection of cells at the injection site. Upon internalisation and translocation to the nucleus, resident cells, such as keratinocytes in the skin and myocytes in muscle tissue, express the vaccine‐encoded antigen and eventually excrete it through apoptosis or exosomes.^[19]^ APCs, such as dendritic cells (DCs), circulate searching for pathogenic structures, detect and internalise the exogenous antigen and introduce it to their endolysosomal degradation pathway. The DCs then progress to the lymph nodes, where they present peptide fragments stemming from the antigen to CD4+ T cells. The activation of CD4+ T cells is achieved through the association of the major histocompatibility class II (MHCII) complex on APCs and the T‐cell receptor on CD4+ cells and supported by further interaction through co‐stimulatory ligand–receptor binding. MHC complexes are a set of genes coding for MHC cell surface glycoproteins, which present pathogen fragments to T cells and thus help the immune system to recognise a threat.^[20]^ CD4+ T cells play a major role in orchestrating the immune response by contributing to B cell priming and the activation of cytotoxic CD8+ T cells.[^21^, ^22^] Furthermore, exogenous antigens can be cross‐presented from transfected apoptotic somatic cells to immature CD8+ T cells through the MHCI pathway, which results in the activation of cytotoxic T lymphocytes (CTL), triggering a strong cellular immune response.^[23]^

In addition to this indirect route, DCs can also be directly transfected by DNA vaccines. The direct route involves the endocytosis and expression of the DNA vaccine in DCs, which results in the parallel activation of CD8+ cytotoxic T cells and distinct CD4+ T helper cells through the binding with MHCI and MHCII, respectively, as well as with the specific co‐stimulating receptors.^[24]^

**Figure 2:**
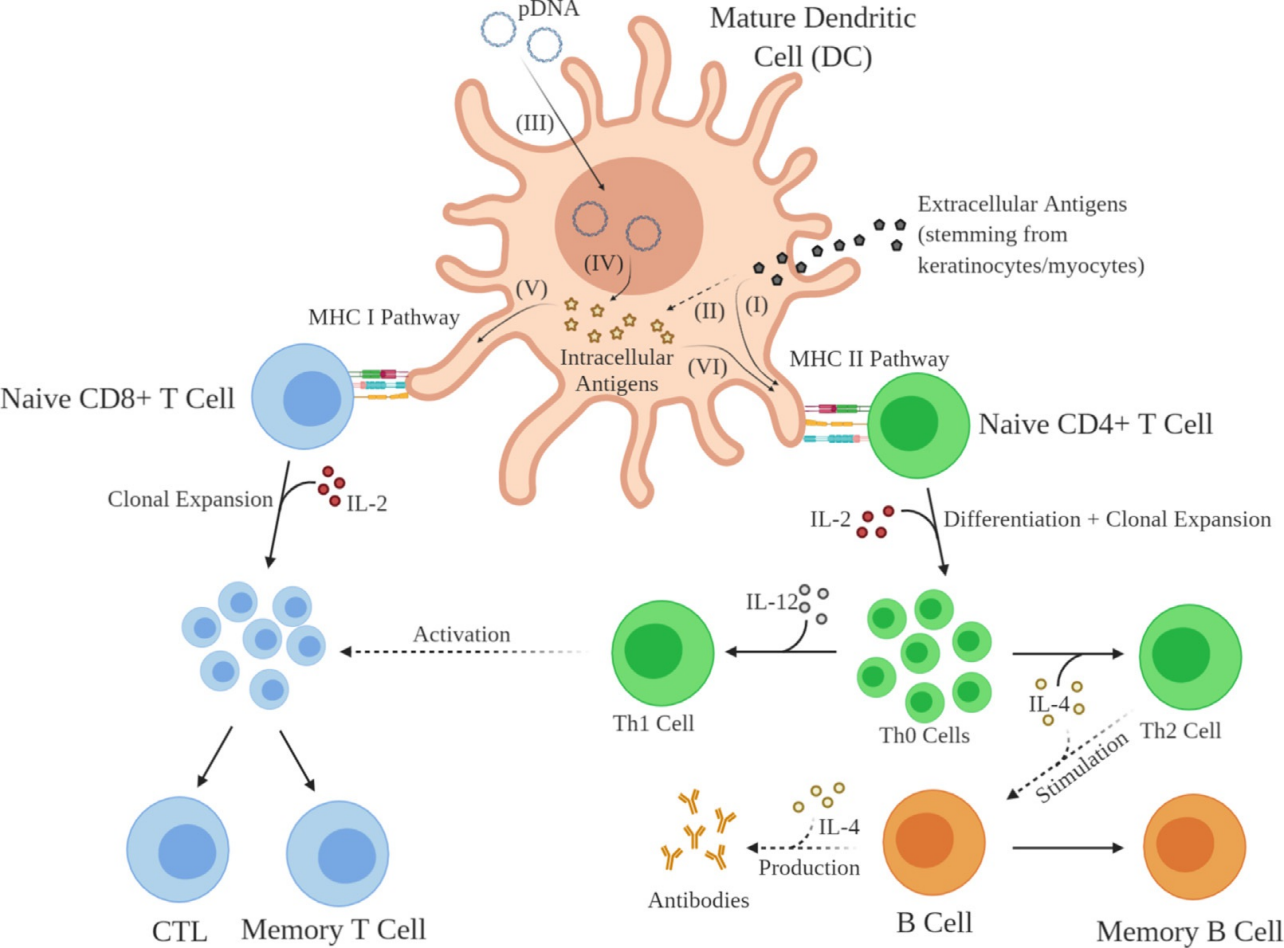
Mode of action of DNA vaccines. Extracellular antigens, stemming from keratinocytes or myocytes, are presented to naive CD4+ T cells through the MHCII pathway (I), which leads to clonal expansion and differentiation to Th0. While the interaction of Th0 with IL‐12 results in the formation of Th1, the binding to IL‐4 promotes the stimulation of B cells, which eventually form antigen‐specific antibodies and memory B cells. Extracellular antigens can also be introduced to the MHCI pathway through cross‐presentation (II). In addition, APCs can be directly transfected with pDNA. After cellular uptake (III), APCs express pDNA (IV) and present the resulting antigen through the MHCI pathway (V) to naive CD8+ T cells, which clonally expand upon the interaction with IL‐2 to form CTL and memory T cells, and to naive CD4+ cells through the MHCII pathway (VI).

Upon activation, immature CD8+ and CD4+ T cells begin to proliferate due to the autocrine production of IL‐2, a T cell growth and differentiation factor (Figure 2). CD8+ T cells clonally expand upon the interaction with IL‐2 to increase the effective amount of cytotoxic, antigen‐specific T cells. CD4+ T cells, however, start to differentiate upon binding to IL‐2 and become T helper cells (Th0). These helper cells produce IL‐2, IL‐4, and interferon gamma (IFN‐γ) and can further proliferate into Th1 and Th2 effector T cells. Th1 cells are responsible for activating the cellular immune system by further supporting the proliferation of cytotoxic CD8+ T cells.^[25]^ In contrast, Th2 cells stimulate the humoral immune response by inducing B cell‐mediated antigen production. In this way, DNA vaccines can trigger both a humoral and cellular immune response, which is a significant advantage over commonly used vaccines.^[26]^ While the humoral immune response targets extracellular pathogens, cellular immunity is responsible for annihilating intracellular pathogens, such as cells infected with viruses or bacteria.

The proliferation into Th1 or Th2 cells can be modulated by changing the mode of administration. While intramuscular administration was shown to mostly induce Th1‐type immune response, intradermal treatment results in a strong humoral, or Th2‐mediated, immune response.^[21]^ Furthermore, this multifaceted mechanism leads the formation of long‐living memory B and T cells to form an integral part of the adaptive immune system, which allows for a faster and stronger immune response after repeated infection with a previously encountered antigen.^[27]^

### 1.2 Design of DNA Sequences

**Figure 3:**
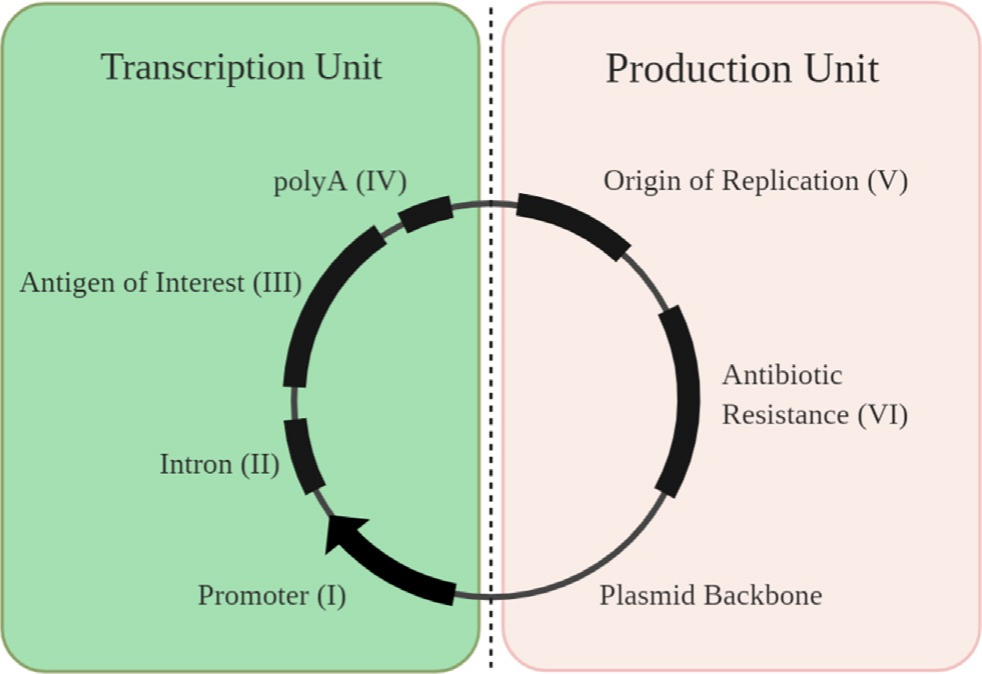
Design of pDNA vaccine sequences. The plasmid can be divided in a production and transcription section. The production unit is responsible for successful replication in the host and consists of an origin of replication and antibiotic resistance gene region. In contrast, the transcription section consists of a promoter region, commonly separated from the antigen of interest sequence by an intron. The sequence is completed by a polyA signal to stabilise mRNA and facilitate nuclear export.

pDNA sequences used in vaccine design are typically comprised of an expression (or transcription) unit and a production unit (Figure 3). The transcription unit consists of a viral‐hybrid or eukaryotic promoter region (I), an intron (II), a sequence that encodes the antigen of interest (III), and a polyadenylic acid (polyA) signal (IV).

The promoter sequence (Figure 3, I) provides a strong binding site for RNA polymerase and thus controls the transcription of the plasmid into mRNA, which is eventually translated into the desired antigen of interest. The most commonly used promoter stems from the human cytomegalovirus (CMV), as it drives high antigen expression levels in various cell lines.^[28]^ However, viral promoters are quickly deactivated through gene silencing and often show only transient gene expression. Therefore, viral/mammalian hybrid‐promoters, such as a combination of CMV and the human elongation factor 1α promoter, can be employed to delay the deactivation effects caused by gene silencing in vitro and in vivo.^[29]^

Typically, an intron sequence (II) is introduced between the promoter (I) and the antigen of interest (III) region on the plasmid. Introns are non‐coding regions in a gene and were found to increase the antigen expression efficacy significantly.^[30]^ The intron is followed by the antigen of interest (III) region, which codes for the desired immunogenic protein. The final element of the transcription unit is a polyA signal (IV), which stabilises the mRNA transcripts and eases the export from the nucleus.[^31^, ^32^]

The production unit consists of an origin of replication (V) sequence and antibiotic resistance genes (VI). The origin of replication is needed to amplify the plasmid in the host cell, and typically consists of a bacterial region, responsible for successful replication and selection in a bacterial host, and an eukaryotic region, which allows for the expression in mammalian cells.^[33]^ The antibiotic resistance genes facilitate the antibiotic‐selected plasmid production in bacterial cultures. However, legislators strongly discourage the use of antibiotic resistance genes in in vivo trials to prevent potential antibiotic resistance of the patient and the integration into the human genome.^[34]^

### 1.3 Delivery of DNA Vaccines

To date, no DNA vaccine has been approved for use in humans. The major challenge for DNA vaccines still resides in insufficient gene expression and low immune system activation. In order to overcome this, research has been focusing on DNA sequence design, vaccine formulation, and the mode of delivery.^[35]^

In terms of delivery, conventional needle‐based delivery approaches can be classified into intradermal, subcutaneous, intravenous, and intramuscular injection with varying depth of skin penetration.^[36]^ Intradermal delivery is considered to be more effective than intramuscular or subcutaneous injection due to the dense DC network present in the dermis.^[37]^ Intravenous application is performed with the goal to deliver DNA plasmids to APCs in secondary lymphoid organs, although with varying effectiveness.^[36]^

Alternatively, DNA vaccines can be delivered via mucosal barriers by, for example, oral, nasal, or pulmonary uptake, which induces local immunity at mucosal sites as well as whole‐body immunity.^[38]^ Mucosal immunisation is regarded to be effective against viruses, as host infection is primarily induced by entering through mucosal surfaces.[^38^, ^39^] Moreover, these methods are particularly user‐friendly, as they do not require needles, specialised equipment, or skilled operators.^[39]^

However, administration of naked DNA through these routes lacks major features desirable for robust immunisation, such as in vivo stability, specific targeting, high cellular uptake, and immune system modulation. Hence, delivery via the skin has been enhanced by use of electroporation, microneedles, or needle‐free delivery systems such as gene guns or biojectors, all of which require specialised equipment.[^36^, ^37^] Delivery efficacy can additionally be increased by combining the pDNA with suitable carriers, which not only improves the plasmid stability, but can also enhance gene expression and immune response. Biopolymers have recently emerged as suitable carrier materials and have significantly increased the potential of DNA vaccine formulations.

## 2 Biopolymers as DNA Vaccine Carriers

**Figure 4:**
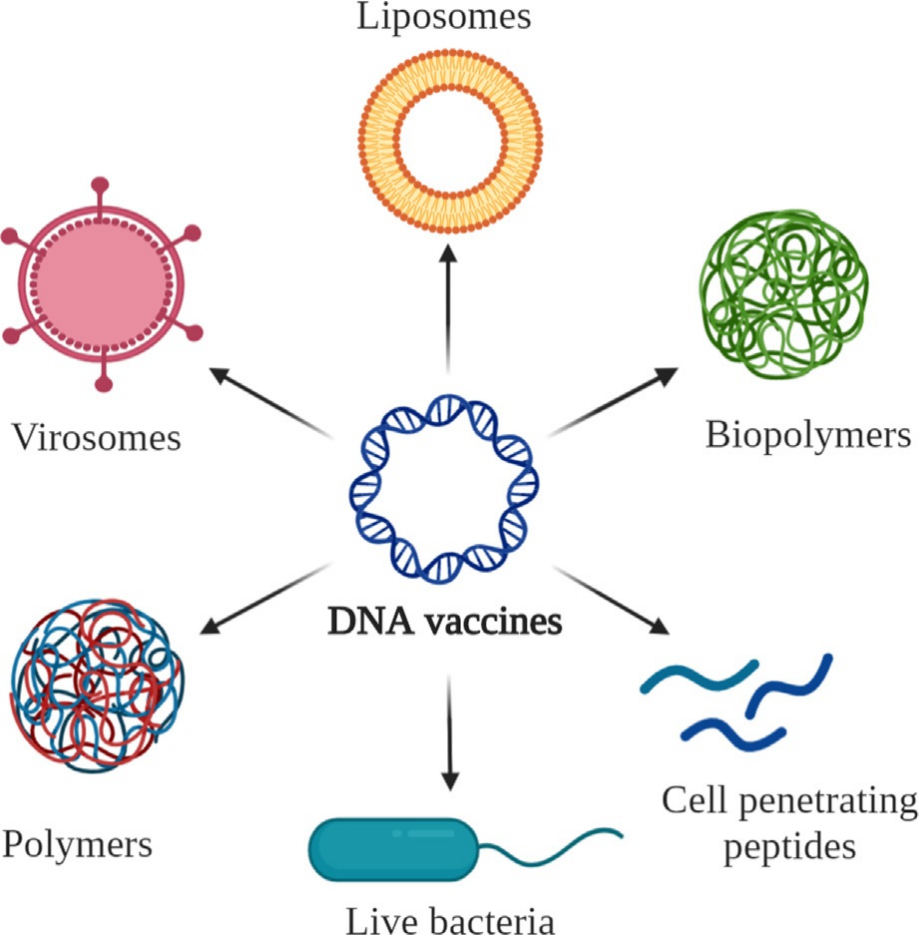
DNA vaccine carriers: liposomes, polymers, virosomes, CPPs, and live bacteria.

Liposomes, polymers, virosomes, cell‐penetrating peptides (CPPs), and live bacteria have been successfully used as DNA carriers (Figure 4).[^35^, ^37^, ^40^] Amongst these, polymers show high physicochemical versatility and low toxicity, provide protection from enzymes that can interfere with the structural integrity of the vaccine, and enable, cost‐effective production.^[41]^ Furthermore, they are more rigid and stable than liposomes and do not pose risks regarding anti‐vector immunity as in the case of virosomes and live bacteria.[^37^, ^42^] An additional advantage of polymers is the ability to design various nanostructures with tuneable size and different surface properties, which have been particularly useful for the design of smart drug delivery systems. In fact, a number of polymer nanoformulations have already been approved for clinical use.[^43^, ^44^] Among polymers for DNA vaccine design, biopolymers (Table 2) are preferred over synthetic polymers due to their biocompatibility, favourable cellular interactions, biodegradability, and often facile production, for example with the help of bacteria or using enzymes.^[45]^ Biopolymers are polymers which are naturally formed by living organisms.^[46]^ In a broader sense, they can be defined as synthetic polymers made from monomer units obtained from living organisms,^[47]^ such as crustaceans, seaweed, and corn.[^48^, ^49^, ^50^] The following sections will give an overview of the most significant advances that emerged from the development of biopolymer carriers and their use in DNA vaccine formulation.

**Table 2:** Properties of biopolymer examples used for pDNA vaccine delivery.

**Polysaccharides**													
*Chitosan* ^*[50–57]*^		*Alginate* ^*[58–61]*^		*Dextran* ^*[62–65]*^		*Chondroitin sulfate* ^*[66, 67]*^		*Hyaluronic acid (HA)* ^*[68, 69]*^		*Pullulan* ^*[70–72]*^			*Pectin* ^*[73, 74]*^
							
+	GRAS^[a]^ status	+	Encapsulates DNA	+	Facile chemical modification	+	Targets CD44 receptors	+	Enables DNA condensation	+	Soluble at wide pH range	+	Used in food industry
+	Cheap production	+	Mucoadhesive and mucopenetrating	+	Prevents accumulation in blood	+	Used in biomedical applications	+	Gel formation with water	+	Used in oral healthcare and pharmaceutical coatings	+	Galactose/arabinose side chains for specific targeting via lectins
+	Easy to functionalise	+	Protects cargo in GI^[b]^ tract					+	Targets CD44 receptor				
+	Good mucosal adhesion (oral delivery)	+	FDA^[c]^ approved										
													
−	Low solubility	−	Burst release of cargo	−	Susceptible to enzymatic degradation	−	Modification required for DNA condensation	−	Modification required for DNA condensation	−	Modification required for DNA condensation	−	Structural complexity limiting reproducibility of results
−	Low transfection efficacy	−	Low encapsulation efficiency when prepared by ionotropic gelation	−	Low cell uptake			−	Short lifetime and quick degradation				
				−	Modification required for DNA condensation								
													
**Proteins**													
*Gelatine* ^*[75, 76]*^		*Albumin* ^*[77, 78]*^		*Listeriolysin O (LLO)* ^*[79–81]*^		*Protamine* ^*[82, 83]*^		*Epsilon poly‐l‐lysine (ϵPLL)* ^*[84]*^		*DC targeting protein* ^*[85, 86]*^			*Zein* ^*[48, 87, 88]*^
							
+	GRAS,^[a]^ used in food industry	+	Stable at wide range of pH and temperatures	+	Disrupts the endosomal membrane	+	Enables DNA condensation	+	Enables DNA condensation	+	Targeted delivery of DNA to DCs	+	Resistant to acidic pH
+	Gel formation with water: easy entrapment of cargo	+	Reduced aggregation with blood components	+	Adjuvant properties: triggers secretion of pro‐inflammatory cytokines	+	DNA endonuclease protection	+	GRAS,^[a]^ used as food preservative	+	DNA condensation	+	Partial resistance to gastric enzymes
						+	Low weight protamines act as CPP						
						+	FDA^[c]^ approved						
													
−	Modification required for DNA condensation	−	Modification required for DNA condensation	−	Modification required for DNA condensation	−	Possibility for aggregation with blood components	−	Possibility for aggregation with blood components	−	Not as versatile (only targets DCs)	−	Modification required for DNA condensation
													
**Bio‐derived polymers**													
*Polyspermine* ^*[89]*^		*Polyarginine* ^*[90, 91]*^		*Polydopamine (PDA)* ^*[92]*^		*Polyglutamate (PGA)* ^*[93]*^		*Poly‐lactic acid (PLA)* ^*[94, 95]*^		*Poly(lactic‐glycolic acid) (PLGA)* ^*[96–101]*^			
					
+	Enables DNA condensation	+	Enables DNA condensation	+	Facile synthesis and modification	+	Facile synthesis and modification	+	FDA^[c]^ approved for clinical use	+	Targets phagocytic cells		
+	Endosomal escape	+	Facilitates cellular uptake	+	Nanoformulation for improved cell uptake	+	Controlled‐release properties	+	Innate gene‐packaging ability	+	Facilitates delivery through biological barriers		
		+	Improves nucleus targeting							+	FDA^[c]^ approved for clinical use		
											
−	Requires crosslinker for synthesis	−	Possibility for aggregation with blood components	−	Modification required for DNA condensation	−	Modification required for DNA condensation	−	Slow degradation	−	Harsh synthetic conditions		
								−	Lack of cell interaction	−	Damage of DNA entrapped within PLGA particles		

[a] Generally recognised as safe (GRAS) is an FDA designation that a substance added to food is considered safe. [b] GI: gastrointestinal. [c] FDA: Food and Drug Administration (USA).

### 2.1 Design of Biopolymer Vaccine Carriers

**Figure 5:**
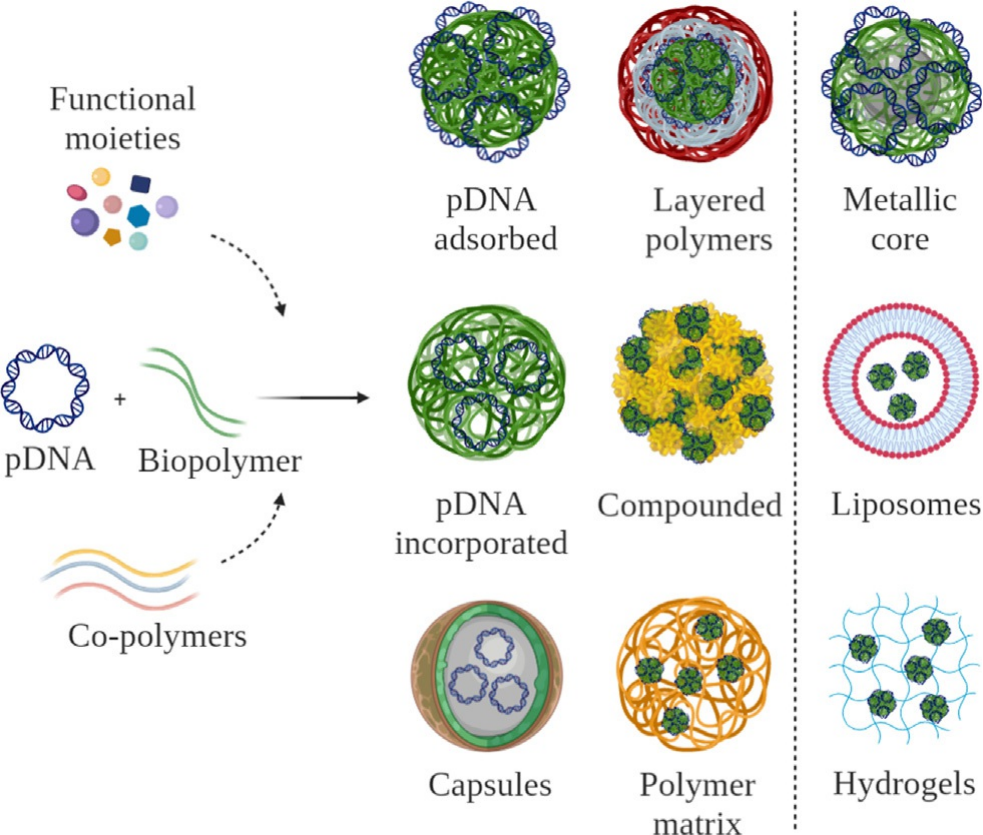
Biopolymer‐based DNA vaccine delivery designs. Nanostructured biopolymers, which can include additional elements such as functional moieties or co‐polymers. Commonly employed designs include NPs with both surface‐adsorbed and incorporated pDNA. Nanocarriers can be further enclosed by polymer layers or incorporated in compounded formulations or polymer matrices. Advanced systems include NPs with metallic cores, the use of liposomes, or hydrogels.

Biopolymeric DNA vaccine systems contain at minimum a core scaffold and the pDNA. The biopolymer core can be modified with functional moieties or co‐polymers to enhance the vaccine delivery (Figure 5). The pDNA can either be adsorbed on the surface of the nanoparticles (NPs)[^101^, ^102^] or incorporated within the core.[^48^, ^103^, ^104^, ^105^] Further modification of these basic designs can be made by addition of polymeric shells,[^51^, ^105^] the deposition of NPs in a polymer matrix,[^105^, ^106^, ^107^] and preparation of compounded NPs composed of two different material domains within the particle.[^108^, ^109^] Moreover, metal‐based NPs, such as gold^[110]^ and iron oxide NPs,[^111^, ^112^] liposomes,[^113^, ^114^, ^115^] and a range of hydrogels have been used in combination with biopolymers to introduce new functionalities.[^99^, ^116^, ^117^]

When designing DNA vaccine carrier materials, different biological barriers need to be taken into account. On an extracellular level, these include the rapid clearance of foreign genetic material from the bloodstream, deactivation through serum proteins, and degradation of DNA through DNases. Once the target cell is reached, carrier‐bound DNA needs to be internalised through the membrane via phago‐, pino‐, or endocytosis. Finally, upon internalisation, the vaccine is required to escape phago‐ or endosomal vesicles and travel across the cytoplasm to reach the nucleus, where DNA needs to dissociate from the carrier to enable the expression of the encoded antigen.^[118]^

In short, the DNA carrier not only needs to ensure the integrity of pDNA, but also needs to be equipped with functional groups that enable stability in biological fluids, prolonged circulation to reach target cells, overcoming of extracellular and intracellular barriers, and safe delivery to the nucleus. This might seem like a formidable challenge, but significant advances have been made in the past decade that took us closer to the rational design of successful DNA carriers. The next sections will give an overview of the most important strategies applied to increase the efficacy of DNA vaccines using biopolymer carriers.

### 2.2 Strategies for Improved DNA Condensation

The association of DNA and the biopolymeric material is crucial to assemble a successful delivery system, as the effective binding results in pDNA protection, and ensures efficient cell uptake. In case of an unstable pDNA immobilisation, premature release of the plasmid can occur, which results in lower transfection efficacies and hence a weaker immune response.^[119]^

By far the most widely exploited strategy that aids pDNA condensation is the use of positive charge. The electrostatic interaction between the negatively charged phosphate group in the DNA backbone and positive charges of the carrier material provides a suitable non‐covalent association strategy for DNA condensation. This phenomenon can be observed in nature, where electrostatic interaction has been adopted for condensation of DNA, for example around the histone or protamine proteins, which are rich in basic amino acid residues.[^120^, ^121^] Numerous biopolymers, among them chitosan,[^101^, ^108^, ^109^, ^122^, ^123^, ^124^, ^125^, ^126^] *ϵ*PLL,[^84^, ^127^] poly‐l‐lysine (PLL),[^67^, ^105^, ^128^, ^129^, ^130^, ^131^, ^132^, ^133^] protamine,[^76^, ^79^, ^80^, ^134^, ^135^, ^136^, ^137^] polyarginine,[^67^, ^90^, ^91^, ^138^] and polyspermine[^89^, ^139^, ^140^, ^141^] (Table 2), bear intrinsic positive charge, making them excellent candidates as DNA condensation agents.

**Figure 6:**
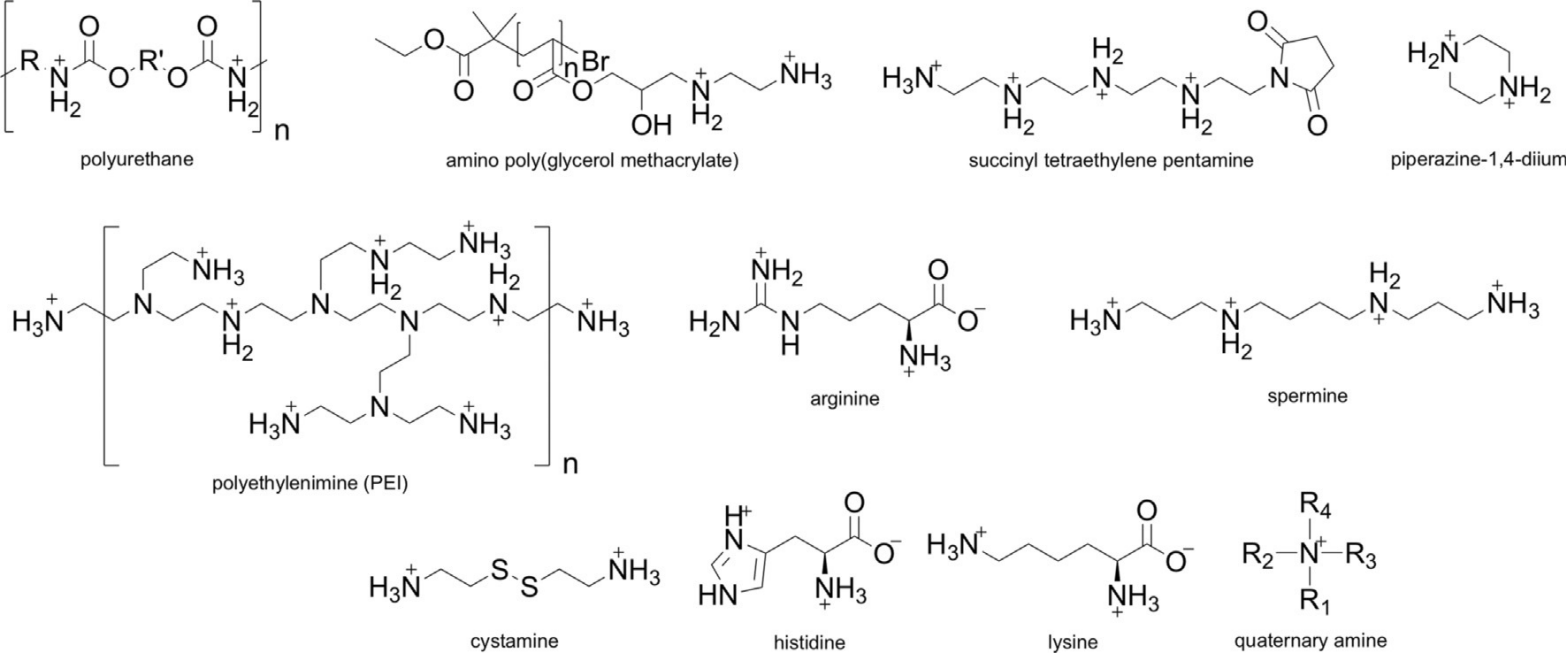
Positive charge facilitates DNA condensation. Commonly used polymers and small organic molecules to introduce positive charge to DNA carriers.

Positive charge can also be introduced to the carrier core by addition of synthetic polymers such as polyethylenimine (PEI),[^64^, ^92^, ^93^, ^111^, ^127^, ^142^, ^143^, ^144^, ^145^, ^146^, ^147^] polyurethane,^[148]^ amino poly(glycerol methacrylate),^[149]^ or positively charged dendrons.[^150^, ^151^] Due to the high density of positive charges, high buffering capacity, and availability, PEI is a widely explored synthetic polyamine in DNA delivery and frequently termed as the “gold‐standard” in polymer‐mediated gene delivery.^[152]^ In addition, covalent functionalisation of biopolymers with positively charged amino acids, peptides, or small molecules (Figure 6) can be used for charge tuning. For example, arginine, histidine, and lysine were employed to functionalise dextran,[^153^, ^154^, ^155^] chitosan,^[51]^ alginate,^[59]^ human serum albumin (HSA),[^78^, ^156^] HA,^[116]^ and chondroitin sulfate.^[157]^ Other small moieties used for biopolymer modification include quaternary amines,[^56^, ^73^, ^158^, ^159^, ^160^] spermine,[^63^, ^161^, ^162^] piperazine,^[163]^ cystamine,^[69]^ and succinyl tetraethylene pentamine.^[164]^

Moreover, the polymer backbone itself can be chemically modified to modulate its intrinsic charge. Chitosan, which is positively charged only at acidic pH, can be trimethylated at its primary amines resulting in a positive charge over a broader pH range.[^55^, ^165^, ^166^, ^167^, ^168^] In case of proteins such as albumin, methyl esterification of carboxyl groups decreases negative charge and renders the overall protein positively charged and well‐suited for DNA condensation.^[169]^

**Figure 7:**
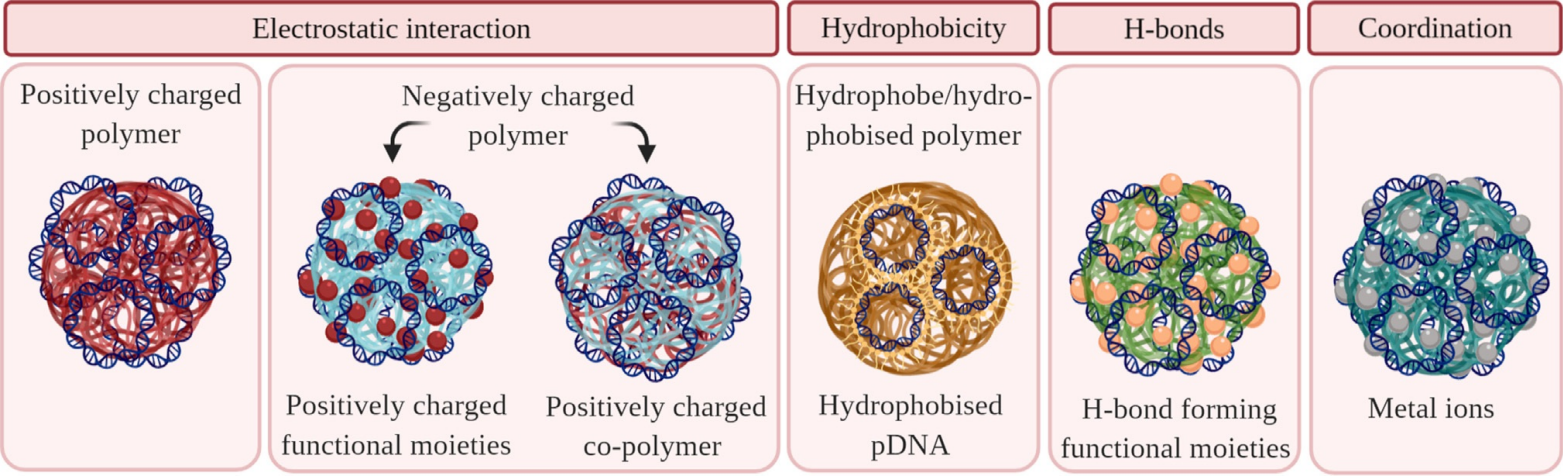
Design of biopolymers for improved DNA condensation. Blue colour corresponds to negatively charged molecules, whereas red colour indicates positive charge.

Alternatively, hydrophobic attraction can be employed to attach pDNA onto the carrier. To achieve this, pDNA can be hydrophobised with cetrimonium bromide^[170]^ or dioleoyl‐3‐trimethylammonium propane.[^98^, ^171^, ^172^, ^173^] The advantage of such an approach is that the carrier material is not only limited to positively charged polymers, but expands the scope to negatively charged or neutral polymers, since electrostatic binding is no longer the driving force for DNA condensation (Figure 7). This approach led to efficient immobilisation of pDNA to 5β‐cholanic acid‐modified chitosan,^[170]^ and 1,2‐distearoyl‐*sn*‐glycero‐3‐phosphoethanolamine‐conjugated HA,^[171]^ as well as for the incorporation of DNA within the hydrophobic core of PLGA particles.[^98^, ^172^, ^173^]

In addition to the use of positive charge and hydrophobic interactions, hydrogen bonding can be exploited to condense DNA.^[174]^ For example, guanidinium moieties in arginine‐containing materials were shown to form hydrogen bonds with phosphate groups in the backbone of DNA and were successfully used for DNA condensation. Furthermore, these groups interact with the cell surface, which significantly improves the cellular uptake.[^174^, ^175^]

Another strategy to enable condensation is the use of coordinating multivalent metals cations that can bind to phosphate groups or nucleobases.[^176^, ^177^] Zinc ions have been employed to aid the interaction between phosphate groups of DNA and two different biopolymeric species, histidine‐conjugated PLL^[178]^ and dipicolylamine‐modified HA,^[179]^ but there is definite scope for use of other ions, particularly those that might be helpful adjuvants, including nickel, beryllium, cobalt, and palladium.^[180]^ In addition to being an excellent coordinating ion, incorporation of zinc ions via histidine has been shown to increase endosomal release increasing the rate of transfection.^[178]^

### 2.3 Improved Stability and Solubility of DNA Biopolymer Systems

Stability and solubility of DNA vaccine formulations are not only essential to ensure the efficacy and safety of the vaccine post administration,^[181]^ but also to enable long‐term storage without compromising the product quality.^[182]^ Notably, stability considerations need to be taken into account when deciding on the route of administration (e.g. oral, parenteral) and the dosage form (e.g. dry powder, suspension) of DNA vaccines.

One of the stability issues commonly encountered with nanoformulations and the production of NP carriers is aggregation, which is especially important for suspension formulations.^[183]^ NPs are characterised by high surface‐area‐to‐volume ratios, resulting in a high surface energy. Consequently, these particles tend to form thermodynamically favourable aggregates with lower overall surface energy, particularly in biological media.[^183^, ^184^]

To prevent aggregation, a surface coating with hydrophilic polymers, such as polyethylene glycol (PEG), can be introduced to the nanoformulation. PEG has been shown to prevent particle interaction and increase their circulation time in blood, thus enhancing the probability of reaching the targeted tissue. PEG surface modification has been demonstrated for PLGA‐,^[98]^ PDA‐,^[185]^ gelatine‐,[^186^, ^187^] polyspermine‐,^[141]^ and PLA‐based^[188]^ DNA carriers.

Shielding against aggregation of positively charged delivery systems can also be achieved using the negatively charged biopolymer chondroitin sulfate.^[67]^ Coating PLL,^[67]^ dendrigraft PLL,^[129]^ poly‐arginine,^[67]^ and protamine^[135]^ NPs with chondroitin sulfate has shown to reduce agglutination with erythrocytes and cytotoxicity associated with highly positively charged delivery systems.^[157]^ Furthermore, Hashimoto and co‐workers showed that functionalisation of chitosan with hydrophilic lactose residues supressed self‐aggregation as well as aggregation of chitosan NPs with serum proteins.^[189]^

Proteins abundant in blood, such as HSA[^78^, ^143^, ^190^] and bovine serum albumin (BSA),^[149]^ known for their role in the transport of various biologically important molecules, ranging from hormones to fatty acids, have also been employed as nanocarriers for gene delivery. As they are naturally present in the blood circulation, unfavourable interactions with other serum proteins are minimised, ensuring low levels of aggregation and favourable pharmacokinetic profiles.^[190]^

In terms of chemical stability, pDNA needs to be well‐protected from degrading DNase enzymes. One way to achieve this is to pack the pDNA within the NP core to block the enzyme access.[^96^, ^191^] However, it was shown that enzyme degradation can also be minimised by adsorption of pDNA on the surface of the carrier.[^77^, ^82^] This is thought to be due to the deformation of pDNA upon binding to nanosized spheres^[192]^ as well as to steric hindrance posed by the carrier core.^[193]^

Considering different routes of DNA vaccine delivery, administrating genetic vaccines via the oral route presents an appealing alternative to intradermal or intramuscular injection due to its non‐invasive and convenient nature, and because of low sterility concerns.^[194]^ In addition, it was shown that oral administration is particularly interesting for local therapy of GI diseases, such as dental caries,^[195]^ colorectal cancer,^[196]^ and inflammatory bowel disease, that is, Crohn's disease and ulcerative colitis.^[197]^ However, oral delivery systems are faced with especially challenging chemical stability issues. Upon oral administration, the nanoformulation encounters the highly acidic stomach environment, which is crowded with gastric enzymes that can degrade the carrier material, thus impeding DNA delivery. This can be overcome by careful design of scaffold materials and DNA‐interacting species. For example, it has been shown that alginate nanospheres can efficiently protect DNA when passing the GI tract and facilitate GI epithelial uptake through superior mucoadhesive and mucopenetrating properties.[^198^, ^199^] Furthermore, tertiary systems comprised of alginate, chitosan, and pDNA were more efficient for in vivo oral delivery than chitosan/pDNA NPs alone.[^200^, ^201^] This is probably due to the dissociation of alginate–chitosan crosslinks at pH 1.5, which results in the formation of a protective layer of insoluble alginate on the carrier surface.^[201]^ Furthermore, the introduction of polycaprolactone and poly(2‐hydroxyethylmethacrylate) to gelatine and chitosan NPs, respectively, proved to be a good strategy to protect DNA cargo from the harsh conditions of the GI tract.[^202^, ^203^]

The protein zein can be used as oral DNA vaccine carrier as well. Its amphiphilic character, owing to its hydrophobic amino acid content of over 50 % and high glutamine content, facilitates aggregation into NPs with hydrophobic cores and hydrophilic outer shells. In acidic environments, such as in the stomach, zein nanocarriers are insoluble, extremely resistant to the low pH, and have been shown to protect their? cargo from enzymatic degradation.[^48^, ^87^] It has been demonstrated that immunisation of mice with chitosan carriers modified with an outer shell of zein leads to higher antibody titers compared to mice vaccinated with particles lacking zein.^[88]^

Finally, it is desirable to develop DNA‐carrier systems stable during storage and transport without extensive cooling, which can be energy demanding and not suitable for low income countries. Designing carriers that enable storage and transport at room temperature is a significant advantage over existing strategies, and has been successfully demonstrated for HA and chitosan‐containing NPs, which remained stable both in lyophilised and liquid form over 12 months at ambient conditions.^[204]^

Closely related to the stability and key to successful DNA vaccine formulations is the solubility of the carrier material. Many biopolymers used for the design of DNA vaccines are intrinsically soluble in aqueous media. However, some commonly used biopolymers, such as chitosan, show only limited solubility at physiological pH. This can be overcome by increasing the hydrophilicity of the polymer backbone through chemical modification, such as the deacetylation of residual acetylated amines,[^205^, ^206^] introduction of carboxy‐methyl moieties to the C6 hydroxy group,[^56^, ^68^, ^207^, ^208^] trimethylation,[^55^, ^165^, ^166^, ^167^, ^207^, ^208^] or addition of larger hydrophilic groups such as *N*‐[(2‐hydroxy‐3‐trimethylammonium)propyl]chloride[^56^, ^159^] and polymeric methacrylates.^[209]^

### 2.4 Improved Cell Uptake

**Figure 8:**
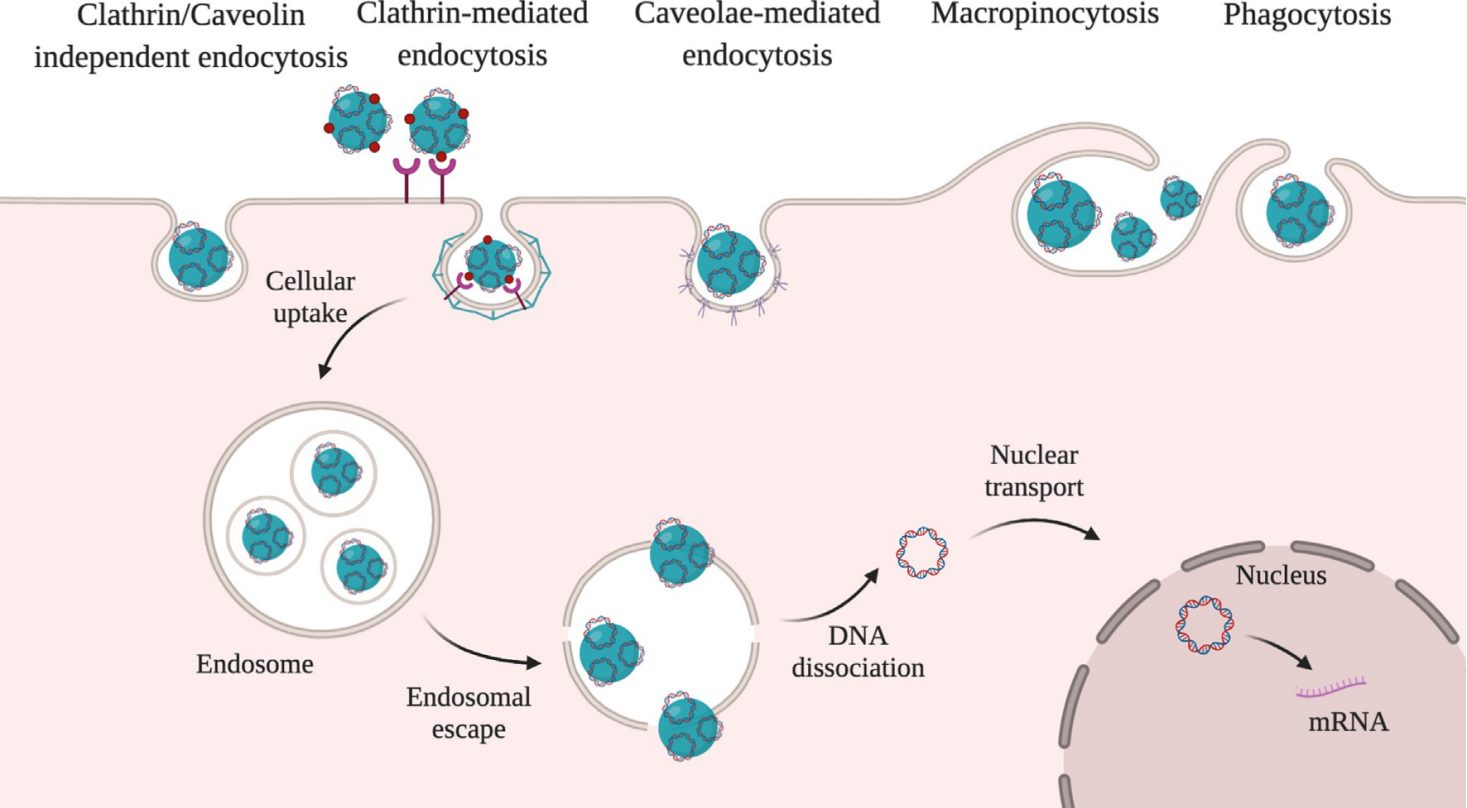
Internalisation of nanocarriers. Internalisation can occur through a number of endocytic pathways: pinocytosis (clathrin‐mediated endocytosis, caveolae‐mediated endocytosis, clathrin/caveolin‐independent endocytosis), phagocytosis, and macropinocytosis. Once internalised, the cargo is entrapped in endosomes and eventually ends up in the lysosome. The pDNA should evade endolysosomal degradation and enter the cytoplasm by endosomal escape to enable pDNA translocation into the nucleus for transcription.

In order to increase the immune response and therapeutic activity of DNA vaccines, efficient delivery and release of the genetic cargo within the targeted cell is crucial. Cellular uptake is one of the most significant steps to ensure the biological activity of vaccines, and it depends on the interaction between the cell membrane and the carriers, which can result in several endocytotic pathways as shown in Figure 8.^[210]^ The internalisation depends on the cell type as cells bear different types and numbers of membrane proteins and lipids, as well as on the physiochemical properties of the carrier.^[211]^ We will not go into details on cellular uptake and release of nucleic acids, since these have been reviewed by Degros et al.,^[212]^ Medina‐Kauwe et al.,^[213]^ and Zhou et al.^[214]^ In the following sections, we will discuss properties of biopolymers that have been reported to promote different routes of DNA vaccine uptake.

#### 2.4.1 Non‐Specific Cell Uptake

Non‐specific, adsorptive endocytosis is promoted by cationic polymers to a higher degree than by negatively charged or neutral molecules.^[152]^ Endocytosis is enabled by adsorption of the positively charged DNA–polymer systems with negatively charged proteoglycans on the outer cell membrane surface.[^174^, ^215^]

**Figure 9:**
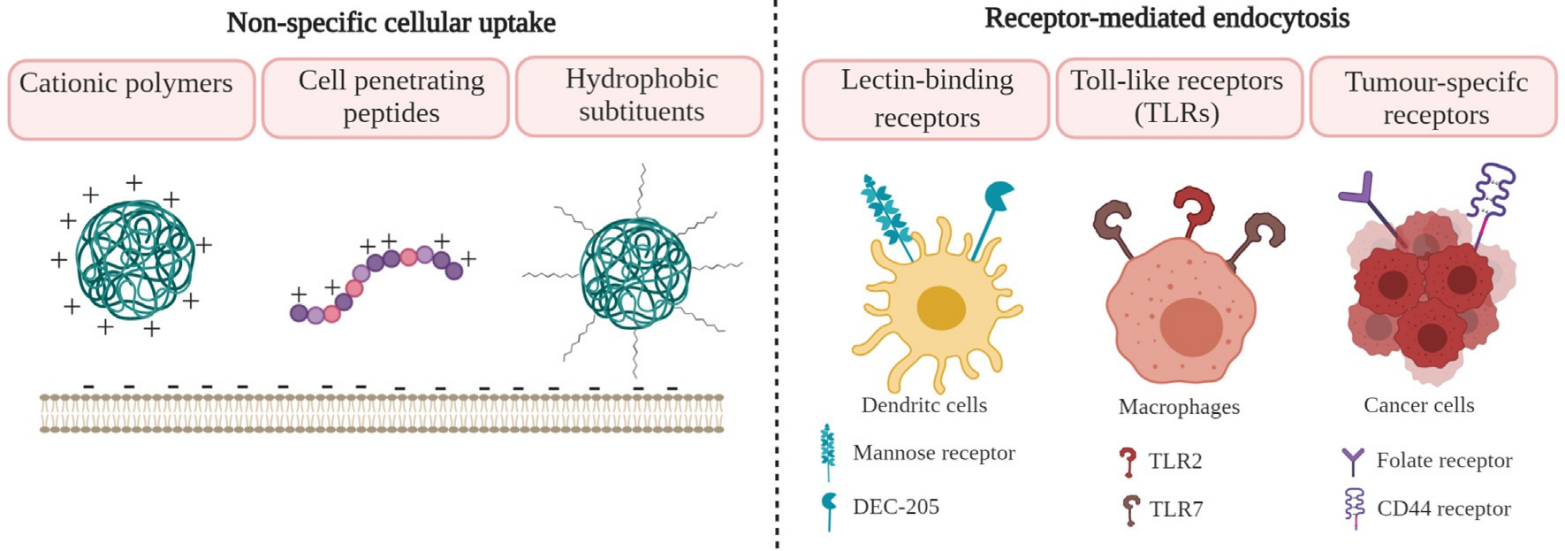
Design strategies to enhance cell uptake of DNA vaccine carriers. Cationic polymers, CPPs, and hydrophobic substituents can promote non‐specific cellular uptake. Targeting lectin‐binding receptors (mannose receptor or DEC‐205) and toll‐like receptors (TLR2 or TLR7) can facilitate accumulation in APCs. Targeting tumour‐specific receptors (folate, CD44) can increase uptake by cancer cells.

In addition to the strategies for the introduction of positive charge reviewed in Section 2.2, non‐specific cellular uptake can be facilitated through the use of CPPs (Figure 9, left).[^78^, ^90^] CPPs are short, positively charged peptides that can translocate across the cell membrane without impairing the cellular integrity and were first discovered in viral proteins. Although the mechanism of action is complex and beyond the scope of this Review, their positive charge plays an important role to facilitate the adsorption onto the negatively charged cell membrane leading to the uptake through several endocytic pathways.[^210^, ^216^] A commonly used CPP is HIV‐1 transactivator of transduction sequence (Tat), which was found to enhance gene transfection efficiency. The introduction of both Tat and arginine‐glycine‐aspartate (RGD) peptide was shown to significantly increase the efficiency of HSA nanovectors.^[78]^ It was also demonstrated that the degree of cross‐linking to the polymer backbone had a pronounced influence on the properties of the carrier. Besides positively charged polymers and CPPs, calcium ions (Ca^2+^) can be used to aid cell internalisation as well as endosomal escape. Ca^2+^ ions mediate several cell processes, including induction of endocytosis. It was demonstrated that the favourable gradient for transport of these ions into the cell (Ca^2+^ concentration is 10^4^ times higher in the extracellular space compared to the cytoplasm) plays an important role in gene delivery.^[217]^ Ca^2+^‐modified alginate‐sulfate NPs were investigated as DNA delivery systems and mechanistic studies indicated the possibility of clathrin‐mediated endocytosis.^[218]^

However, it was reported that large amounts of positively charged polymers, especially if bearing high charge density, can cause cytotoxicity^[219]^ and promote nonspecific interaction with negatively charged serum proteins and subsequent clearance by the reticuloendothelial system.^[220]^ That indicates the importance to control the amount of positive charge and charge density.

One approach to reduce positive charge without reducing non‐specific cellular uptake includes introduction of hydrophobic moieties such as phenylalanine^[102]^ or oleoyl groups^[68]^ to the positively charged polymer backbone. Although this was shown to decrease the toxicity and enhance cellular uptake through hydrophobic interactions with the cell membrane,^[221]^ careful introduction of such modifications is needed in order to avoid significant decrease in solubility and the stability of the system.

#### 2.4.2 Target‐Specific Cell Uptake

Targeting ligands can enhance accumulation of the carrier system in a specific tissue or cell type, minimise non‐specific uptake, and facilitate internalisation. Small molecules, such as folate,[^141^, ^148^, ^163^, ^222^, ^223^] alendronate,^[224]^ lactose,^[189]^ mannose,[^113^, ^225^, ^226^, ^227^] and TLR7 agonist,^[208]^ as well as peptides,[^207^, ^228^, ^229^] oligonucleotides,^[187]^ and polymers[^68^, ^129^, ^142^, ^172^, ^173^, ^222^] have been introduced to biopolymer‐based nanovectors in order to improve targeting of specific receptors (Figure 9, right). In addition to the choice of ligand, control over surface density is essential to achieve high targeting efficiency and internalisation.

Specific delivery and enhanced uptake of DNA vaccines into APCs has been associated with effective immunisation. Lectin‐binding receptors, such as mannose receptors CD206 and DEC‐205, are abundantly expressed on the surface of APCs, including macrophages and DCs,[^225^, ^226^, ^227^, ^230^] and introduction of mannose enables preferential uptake of APCs.[^113^, ^225^, ^226^, ^227^]

The DEC‐205 receptor is particularly interesting, as it initiates MHCI and MHCII pathways by antigen endocytosis, leading to the stimulation of both CD4+ and CD8+ T cells.^[231]^ To enable DEC‐205 targeting, Suresh et al. fused anti‐DEC‐205 antibody to pDNA‐loaded chitosan and designed a DNA vaccine against severe acute respiratory syndrome coronavirus (SARS‐CoV) nucleocapsid protein.^[228]^ This carrier has demonstrated that targeted delivery to nasal DCs is a potent strategy to achieve enhanced immunogenicity of a low‐dose DNA vaccine.

In addition to lectin‐binding receptors, TLRs have been explored for macrophage targeting. Modification of chitosan NPs with TLR agonists, TLR‐7^[208]^ and TLR‐2,^[207]^ has significantly increased IL‐8 levels in THP‐1 macrophages compared to bare chitosan NPs.

As mentioned in the introduction, DNA vaccines represent a promising strategy to induce a specific long‐term immune response. This is particularly interesting for the induction of an immunological memory and systemic immune response in cancer treatment.^[232]^ Besides targeting receptors on APCs, different cancer types can be treated by induction of specific CTLs. Namely, folate and CD44 receptors are significantly overexpressed receptors on several tumour cells compared to normal cells. Therefore, introducing folic acid to chitosan,^[223]^ polyspermine,^[141]^ chondroitin sulfate‐PEI,^[222]^ and dextran[^148^, ^163^] led to enhanced immunisation in hepatocellular, lung, and ovarian cancer, respectively. Biopolymer scaffolds, such as endogenous polysaccharides, HA, and chondroitin sulfate, specifically bind to CD44 receptors.

### 2.5 Designing Carriers that Enable Endosomal Escape

A major challenge drug and gene delivery systems are facing is their entrapment in endosomes. Endosomes are organelles responsible for intracellular sorting and contain numerous enzymes. If the genetic material remains entrapped in the endosome, it is degraded by lysosomal proteases, which ultimately results in low transfection efficacy. In order to escape endolysosomal degradation, several mechanisms are involved in this process, such as pore formation in the endosomal membrane, the pH buffering effect, and the fusion into the lipid bilayer.^[233]^

Throughout the maturation of the endosomes, the pH decreases from physiological pH 7.4 to pH≈6.5 in the early endosome, pH≈6.0 in the late endosome and pH≈5.0 in the lysosome due to the activity of membrane‐bound ATPase pumps (proton pumps), which pump protons across the endosome and lysosome membrane into the vesicle interior through ATP hydrolysis.^[234]^ The presence of cationic polymers, such as PEI,[^91^, ^142^, ^144^, ^147^, ^152^, ^235^] polyamidoamine,^[151]^ succinyl tetraethylene pentamine,^[164]^ spermine,[^89^, ^139^, ^140^, ^141^, ^161^, ^162^, ^236^] and imidazole‐containing molecules, such as histidine,[^83^, ^90^] can all lead to endosomal rupture via the “proton‐sponge” effect. This effect is associated to the large buffering ability of these molecules due to proton binding, which leads to more protons being pumped. Consequently, this results in accumulation of chloride ions and water, osmotic swelling, and ultimately rupture of endosomes. Even though this mechanism has been extensively used to explain the endosomal rupture, it has been heavily debated.^[234]^ Nevertheless, molecules with high buffering capacity have been shown to play an important role in enabling endosomal escape and have been employed to enable the release of genetic material. For example, Cheng et al.[^153^, ^154^] have designed dextran nanocarriers containing histidine‐rich peptides as a promising material for safe and efficient gene therapy. In their study, dextran was grafted with arginine–histidine peptides (R_*x*_H_*y*_), and low cytotoxicity as well as high gene expression were achieved by using low molecular weight dextran carriers with a high degree of substitution. The presence and ratio of histidine was important both for the DNA condensation and the control of endosomal escape, while arginine residues were primarily used for DNA condensation and enhanced cell uptake. This and other studies[^64^, ^83^, ^90^, ^139^] demonstrated the advantage of imidazole substituents and indicated a possible route to designing efficient DNA vaccine carriers.

In addition to functional groups with high buffering capacity, it was also shown that endosomal escape can be enhanced in the presence of molecules able to penetrate lysosomes (lysosomotropic agents), such as chloroquine,[^237^, ^238^] cationic lipids,^[239]^ and membrane‐disrupting peptides.[^159^, ^240^] The latter are particularly interesting as they are mainly derived from viral and bacterial vectors, which developed efficient strategies to escape endosomes.^[233]^ For example, membrane‐disrupting peptides, such as haemagglutinin subunit 2 (HA2),^[240]^ a fusogenic peptide from influenza virus, and LLO, a cholesterol‐dependent toxin produced by *Listeria monocytogenes*,^[241]^ have been used to modify pDNA delivery systems and enhance delivery into the cytosol of the target cells. Although their mode of action is different, they both result in endosomal escape. While HA2 undergoes conformational change during acidification, which enables fusion trough the endosomal membrane, LLO is active at low pH but is degraded in the cytosol and enables endosomal escape through the formation of pores in lipid bilayers.^[80]^

### 2.6 Improved pDNA Release and Delivery to the Nucleus

Dissociation of the pDNA from the nanocarrier is one of the most important steps in achieving higher transfection efficacies. Efficient release can be achieved either by masking the strong electrostatic interaction of pDNA and the carrier or by introduction of a stimuli‐responsive degradation system.

Masking of the positive charge is probably the simpler, but less controllable route and can be achieved by introducing a second polymer to the nanocarrier. For example, it was reported that incorporating polyanionic polymers, such as alginate[^200^, ^201^, ^242^] and poly(γ‐glutamic acid),[^108^, ^109^] or a negatively charged protein like α‐casein,^[243]^ into chitosan nanostructures can reduce the strength of the interaction between DNA and the particles, facilitating release and increasing transfection. However, it should be noted that this could also lead to a loss of DNA as it is transported to the cell of interest.

Studies have also demonstrated that the net positive charge can be decreased by grafting hydrophobic molecules such as l‐phenylalanine^[102]^ or by addition of flexible bulky groups, such as pullulan.^[64]^ Additionally, Xu and co‐workers integrated a dendritic lipopeptide, a charge‐reversible polymer, and an APC‐targeting material into a DNA vaccine delivery system through layer‐by‐layer assembly.^[51]^ They used poly(allylamine hydrochloride)‐citraconic anhydride, a commonly used charge‐reversible polymer, which can be hydrolysed in mild acidic environments in endosomes, resulting in the destabilisation of the carriers and release of pDNA. Compared to the traditionally used cationic polymer, PEI_25k_, the carrier system based on the charge‐reversible polymer resulted in lower toxicity and eight‐fold higher transfection efficiency.

However, a more controllable route to pDNA release is the use of stimuli‐responsive linkers. In DNA vaccine systems, stimuli‐responsive disintegration of the carrier is mainly achieved by incorporation of disulfide bonds and pH‐sensitive bonds.

The disulfide bond (‐S−S‐) can be cleaved into thiols (‐SH) in the presence of intercellular glutathione.^[244]^ The concentration of glutathione is around 100–1000 times higher in several intracellular compartments compared to the extracellular environment (2–10 mm compared to 2–20 μm), which contributes to the efficient cleavage of disulfide modified polymers.^[245]^ This strategy was employed to facilitate unpacking of pDNA from polymer carriers such as dextran,[^148^, ^163^] HA,^[69]^ polyarginine,^[90]^ and LLO,[^79^, ^80^] which were modified with positively charged groups via disulfide bonds.

In addition to disulfide bonds, acid‐degradable ketal esters,^[89]^ diacrylate cross‐linkers,^[141]^ and bis‐amide bonds^[139]^ were used to improve the release of DNA from polyspermine gene delivery systems in acidic environment.

Furthermore, Liu and co‐workers have reported the dissociation of pDNA from dextran–quantum dots^[246]^ enabled by cleaving the C=N bond in Schiff bases in acidic conditions (pH<6.5).^[247]^

In another study, Wang and co‐workers have designed a PDA–PEI nanovector bound to PEG–phenylboronic acid via a pH‐responsive boronate‐ester bond.^[185]^ They have shown that the complex remained stable at physiological pH but could be cleaved after internalisation in endosomes. Additionally, near‐infrared (NIR)‐light irradiation and good photothermal conversion of the carrier system has resulted in quick endosomal release. In response to the acidic pH within cancer cells and the NIR light irradiation, the nanovector was able to overcome multiple barriers and result in efficient gene delivery.

Once released, pDNA needs to enter the nucleus to be transcribed for successful DNA vaccination. To aid the nuclear transport of the genetic material, carriers can be additionally modified with a nuclear localisation signal (NLS). NLS is a short peptide with high content of positively charged lysines or arginines, derived from eukaryotic nuclear proteins and viral proteins, which can efficiently mediate intranuclear transport.^[248]^ Guan et al. introduced NLS derived from simian virus 40 (SV40) large T antigen to a cationic HSA–DNA system, significantly increasing its gene expression efficacy in vitro and in vivo.^[190]^

Similarly, protamine, an arginine‐rich protein commonly used for DNA condensation and membrane translocation, has been shown to aid the nucleus uptake when attached to biodegradable anionic polymers such as chondroitin sulfate^[135]^ or hydrophobic moieties such as cholesterol.^[83]^ This increase in nuclear transport is attributed to the presence of NLS‐like regions consisting of four to six arginine repeats.

### 2.7 DNA Vaccine Delivery Materials with Immunostimulatory Effects

As outlined in Section 1.1., immunostimulants, such as IFN‐γ, IL‐2, or IL‐4, play an important role during an immune response, i.a. through promoting T cell differentiation. This is the reason why vaccine formulations often contain adjuvants. Adjuvants are organic or inorganic vaccine additives, for example, aluminum salts in hepatitis vaccines or monophosphoryl lipids in shingles vaccines, and are employed to trigger a stronger immune response, for example through stimulating the secretion of immunostimulants.^[249]^

Adjuvants have also been employed to boost the immunogenicity of DNA vaccines. For example, Jiang et al. reported that modifying chitosan NPs with methacrylate‐based polymers resulted not only in a significant stabilisation of DNA, but also led to higher antibody levels and IFN‐γ secretion compared to the administration of naked DNA.^[209]^

Further to these findings, Yue and co‐workers reported that the introduction of CpG motifs to chitosan‐NP‐carriers increased T‐cell proliferation, the production and release of IFN‐γ, IL‐2, as well as IL‐4.^[250]^ The CpG motif is a well‐studied adjuvant in gene delivery and consists of a short, synthetic single‐stranded DNA sequence, which contains mostly cytosine and guanine building blocks. In nature, the unmethylated CpG motif can be found in bacterial genomes, but it is very rare in the vertebrate genome. Thus, when brought into the organism of a vertebrate, the foreign CpG DNA is recognised as an invading species, which results in a strong immune response. In a nutshell, CpG represents a pathogen‐associated molecular pattern (PAMP), which is recognised by a specific pattern recognition system in TLR‐9 of APCs.^[251]^

Interestingly, not only the addition of adjuvants but also the size of the carrier complex plays a role in provoking an immune response. This phenomenon was observed in various inorganic and biopolymeric delivery systems. However, no universal, linear size–immune‐response relationship was derived, indicating that other factors, including shape, chemical composition, surface modification, and charge of the carrier, contribute, too.^[252]^ While Yue and co‐workers found that smaller CpG‐modified chitosan NPs triggered a stronger immune response than larger particles,^[250]^ Kim et al. explored the potential of monodispersed polypyrrole NPs and discovered that medium‐sized NPs (60 nm in diameter) caused a stronger immune response than their smaller and larger counterparts.^[253]^ These studies point towards the need for a rational design approach that aims to address different factors related to stability, solubility, transport, and immune system response of DNA vaccines.

## 3 Conclusion and Future Directions

DNA vaccines have been developed as a suitable alternative to conventional vaccines. Despite not being approved for human use yet, numerous DNA vaccine formulations have been proposed and used in clinical trials to tackle, i.a., viral and bacterial infections, parasites, as well as cancers. There are several advantages of DNA vaccines that should prompt more studies in the field. DNA vaccines are cheaper to make and easier to formulate than conventional vaccines. They are based on the expression of antigens, which can be presented to immune cells, making them versatile and adaptable to a range of diseases. However, pressing challenges remain, including the difficulty of administering genetic material precisely and safely as well as the low immunogenicity of the first formulations. Some of these obstacles have been overcome by using DNA nanocarriers, based on polymers, lipids, and inorganic materials. Biopolymer‐based nanomaterials are particularly promising carrier candidates due to their intrinsic biocompatibility, biodegradability, sustainable availability, and manifold possibilities to modulate their physicochemical and biological properties by adjusting the size, chemical composition, or surface functionalisation. These materials can be further modified to improve their solubility and stability, ability to immobilise DNA, cellular uptake, intracellular release, as well as immunogenicity.

The promising initial advances of biopolymer‐based DNA vaccines, particularly for the treatment of cancers, are reassuring, but the major challenge of the field, the insufficient immune response in humans through administered DNA, abides. Hence, the field will need to focus on translating the promising results of preclinical studies, commonly conducted in small mammals and non‐human primates, to efficient human therapies. This is often very difficult due to the significantly different structure of the immune system in humans and animals. Furthermore, we expect that particularly for complex diseases, like cancer, clinicians will embrace an approach based on the combination of therapies, such as checkpoint inhibition, cell therapy, and DNA vaccines to address the issue of low immunogenicity in the future. Recently, combinational approaches have also been employed in dealing with coronavirus infections, wherein antivirals and anti‐inflammatory agents are being used to counter viral replication and respiratory distress, respectively. Administration of vaccines alongside these conventional therapies will effectually counter the virus that enters the body.

A development we will need to see in the next few years is a more efficient identification of biomarkers, which will enable development of diagnostic strategies and provide us with the information on patients most likely to benefit from use of the DNA vaccine approaches. A more nuanced and holistic understanding of immunological markers and their correlation to vaccine efficacy will inform us about polymer design and its subsequent delivery strategy.

Finally, scale up of vaccine‐based delivery systems with focus on reproducibility and stability is another crucial aspect to consider both during the carrier material design and implementation in the clinic. We believe that in this context, biopolymers will emerge as sustainable and scalable material of choice for the formulation of DNA and mRNA vaccines, as their monomers are readily available and their production is cost‐effective. The next few years will see more research in the field of biopolymer‐based DNA vaccines to increase their in vivo efficacy and ultimately enable their application in the clinic.

## Conflict of interest

The authors declare no conflict of interest.

## Biographical Information

*Christoph Franck holds BSc and MSc degrees in Chemistry and Polymer Chemistry from Karlsruhe Institute of Technology, where he worked under the supervision of Prof. Christopher Barner‐Kowollik on the development of λ‐orthogonal photoligation strategies employing visible light. In 2018, he received an MRes in Sensor Technologies and Applications from University of Cambridge. He is a SensorCDT and AstraZeneca PhD student developing novel “Bio‐Hybrid Nanomaterials for Vaccine Formulation” in the group of Dr. Ljiljana Fruk at the University of Cambridge*.

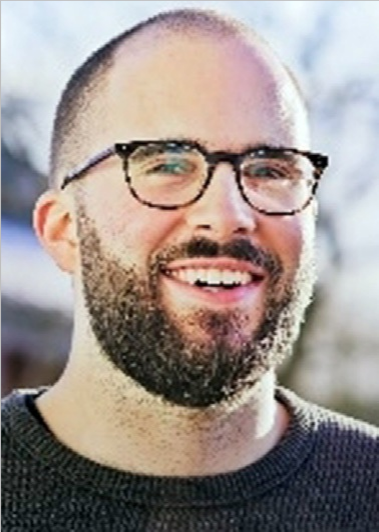

## Biographical Information

*Luise Fanslau received her BSc degree in Molecular Biotechnology from Heidelberg University, Germany, where she was working on photoswitchable DNA in the group of Prof. Dr. Andres Jäschke. Currently, she is an MPhil Biotechnology student at the University of Cambridge, where she is doing research on novel DNA vaccine delivery carriers in the group of Dr. Ljiljana Fruk. She is a scholar of the German Academic Scholarship Foundation*.

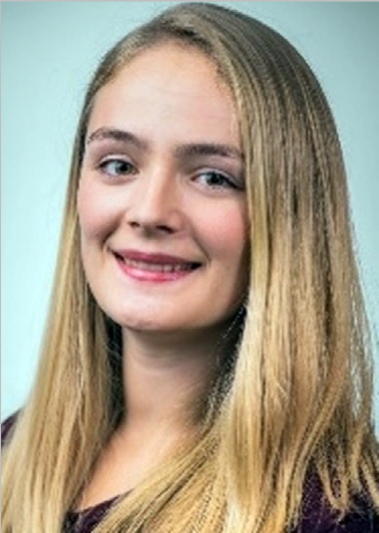

## Biographical Information

*Andrea Bistrovic Popov received her PhD in Organic Medicinal Chemistry from the University of Zagreb in 2018, where she worked on the synthesis of novel purine isosteres as anticancer agents using green chemistry approaches. She is currently a Postdoctoral Research Associate with Dr. Ljiljana Fruk at the Department of Chemical Engineering and Biotechnology, University of Cambridge. Her research is focused on the design of polymer nanocarriers for drug delivery in hard to treat cancers*.

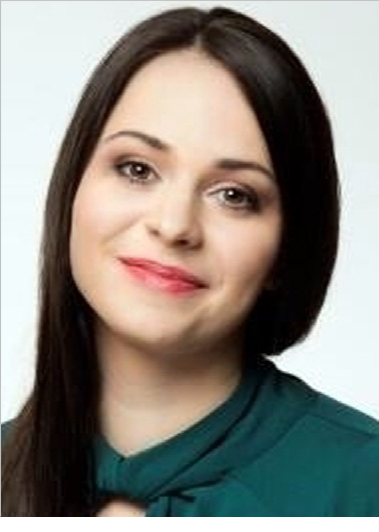

## Biographical Information

*Puneet Tyagi is a senior scientist at AstraZeneca, based in Maryland, USA. He received his PhD in pharmaceutical sciences from the University of Colorado. His area of research is biologics formulation development, with a focus on sustained delivery systems and oral delivery of biologics. Puneet is co‐inventor of several U.S. and international patents and has published extensively in the field of drug delivery. He is a member of various professional organisations including the American Association of Pharmaceutical Scientists (AAPS), where he is serving as a steering committee member of the AAPS Nanotechnology Community since August 2015*.

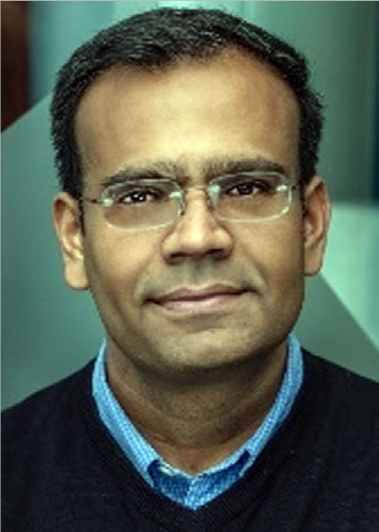

## Biographical Information

*Ljiljana Fruk is an associate professor of Bionanotechnology at the Department of Chemical Engineering and Biotechnology, University of Cambridge. She studied chemistry at the University of Zagreb, and completed her PhD at the University of Strathclyde, Glasgow, working on DNA detection by advanced spectroscopies, such as SERRS. After postdoc research on DNA nanostructuring and artificial enzyme design at the University of Dortmund, she was a group leader at Karlsruhe Institute of Technology before joining Cambridge. Her research focuses on nanostructured biomaterials for use in photocatalysis and targeted drug delivery*.

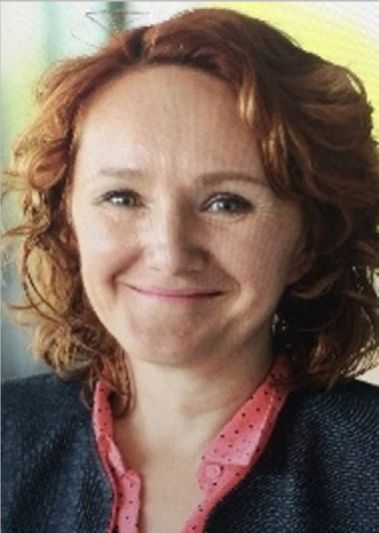
